# The Absence of International Standardized Quality Criteria in Doctorate Programs in Surgery: A Survey Study

**DOI:** 10.1177/23821205251389679

**Published:** 2025-11-17

**Authors:** Núria Llorach-Perucho, Manuel Pera, Eloy Espín-Bassany, Joan-Francesc Julián-Ibáñez, Juan Morote-Robles, Natalia Amat-Lefort, Álvaro Serra-Gómez, Luis Grande, Salvador Navarro-Soto, Xavier Serra-Aracil

**Affiliations:** 1 203277 Parc Taulí Hospital Universitari, Sabadell, Spain; 2 Autonomous University of Barcelona, Cerdanyola del Vallès, Spain; 3 16810 Vall d'Hebron University Hospital, Barcelona, Spain; 4 16514 Hospital Germans Trias i Pujol, Badalona, Spain; 5 4496 Leiden University, Leiden, the Netherlands; 6 16548 Hospital del Mar, Barcelona, Spain; 7 16719 Universitat Autonoma de Barcelona, Barcelona, Spain; 8 General and Digestive Surgery Service, Parc Tauli Institute for Research and Innovation I3PT-CERCA, Parc Tauli University Hospital, Sabadell, Spain

**Keywords:** PhD quality, doctorate program, surgery, excellence-PhD (e-PhD), global variability

## Abstract

**Purpose:**

A doctorate degree in surgery is awarded by universities in recognition of high-standard academic research. This study explores the global heterogeneity of PhD programs in surgery and evaluates them using a standardized rating scale.

**Method:**

A cross-sectional survey was distributed electronically to surgical doctoral programs worldwide. A 25-point rating scale was developed to assess program quality across domains such as dissertation requirements, number and type of publications, journal quartiles, and authorship position. Programs achieving ≥15 points were classified as “excellence-PhD” (e-PhD). Scores were compared across world regions and by university ranking (Shanghai Ranking).

**Results:**

A total of 949 PhD programs from the 193 United Nations member countries were contacted. Completed questionnaires were returned by 187 departments (response rate 19.7%) from 52 countries. Most departments, 138 out of 187 (73.9%) lacked clear requirements for dissertations based on a single research project, while more explicit criteria existed for thesis by publications: originality 77 out of 187 (41.2%), journal metrics 126 out of 187 (67.9%), and candidate authorship. Program scores showed wide heterogeneity, with higher scores more frequently associated with universities ranked higher in the Shanghai classification. Regional differences were also noted in funding opportunities and evaluation processes.

**Conclusions:**

Global PhD programs in surgery demonstrate substantial variability in structure and quality, as measured by a newly developed rating scale. While the scale shows promise in identifying high-quality (e-PhD) programs, its practical application may be limited by response bias and differing academic norms. Nonetheless, these findings offer a framework for benchmarking and improving doctoral training in surgical research globally.

## Introduction

Accomplishment of a doctorate degree in Surgery is an indispensable requirement for progression in the academic field, with the highest achievement of the rank of full professor in Surgery. However, the conception and requirements of a PhD in Surgery vary substantially across institutions, and in all cases PhD studies demand significant scientific effort from candidates. For uniformity in this study, we considered a PhD synonymous with a university doctoral degree in Surgery.

At the international level, doctoral education shows wide heterogeneity in teaching, supervision, assessment practices, and research demands, even within the same country.^1,2^ In Spain, for example, doctoral programs are regulated by the Royal Decree law 99/2011, which provides only broad quality guidelines.^3^

Studies confirm that the research environment (including active, research-oriented supervisors, institutional infrastructure, and academic cultures) significantly influences PhD student productivity, more so than prior academic performance. For instance, students guided by experienced supervisors in priority research areas had higher publication counts, impact, and lower attrition rates, while funding also played a decisive role in PhD outcomes.^4^ Additionally, productive doctoral environments foster not just publication volume but also scientific connectivity and networks.^5^ Institutions with strong research cultures have been identified as key settings propitious to doctoral output.^6^ Emerging evidence also highlights the role of support networks (from peers, mentors, to family) as critical enablers of productivity during the PhD journey.^7^ Notably, funding deficiencies have been associated with increased attrition from MD-PhD programs in the United States.^8,9^

Global reviews of postgraduate surgical education confirm that marked disparities exist between regions. In low- and middle-income countries, doctoral and postgraduate surgical programs are often heterogeneous, adapted to local needs, and lack standardized international criteria, leading to significant variability in training, supervision, and assessment.^10–13^ In contrast, high-income countries also show discrepancies—though of smaller magnitude—particularly in program duration, fellowship requirements, and implementation of competency-based models.^8,9,14^ For example, curricula in the United Kingdom and Australia emphasize longer training and stricter operative requirements than those in other developed countries.^14^

With respect to research outputs, publication-based dissertations have gained prominence and been linked to higher scientific productivity and impact.

As for research, interestingly, a study at The Charité-University Medicine Berlin in which publication-based thesis were supported since 2000 and included in the doctoral regulations since 2005, showed an increase in the number of publications per doctoral student from 0.78 to 1.34 and an average impact factor from 2.42 to 3.62 in a comparison of >250 thesis for each year completed in 1998, 2004, and 2008.^15^ In a study of 841 PhD theses from the Department of Clinical Medicine, University of Copenhagen, concluded in 2013-2017, a total of 2845 manuscripts were embedded in the theses, with a mean (SD) of 3.4 (0.8) manuscripts/thesis.^16^

A Data-Based Assessment of Research-Doctorate Programs in the United States provides datasets that can be used to assess the quality and effectiveness of doctoral programs based on measures important to faculty, students, administrators, funders, and other stakeholders,^17^ but an international analysis of programs focused on obtaining a PhD in surgery has not been previously reported.

Previous research has documented important variability in postgraduate surgical education across regions. In low- and middle-income countries, doctoral and surgical training programs are often heterogeneous and lack standardized criteria, leading to disparities in supervision, funding, and assessment practices.^10–12^ In high-income countries, although programs are generally better resourced, differences persist in duration, fellowship requirements, and implementation of competency-based curricula.^8,9,14^ Moreover, studies have shown that research environments, including quality of supervision and institutional culture, play a decisive role in doctoral productivity, while funding deficiencies are linked to higher attrition rates.^4,5^ Publication-based dissertations, increasingly adopted in Europe, have been associated with higher research output and impact compared with traditional thesis formats.^15,16^

Despite growing international interest in doctoral education in medicine and surgery, no prior study has systematically analyzed the variability of doctoral programs in Surgery across countries. Existing evidence is limited to national studies, such as one in Spain showing marked variability in research project quality, authorship position, and journal quartiles required for the PhD degree.^2,3^ This represents a critical research gap: heterogeneity in doctoral requirements may influence not only the quality of research training but also the comparability of academic credentials worldwide.

The present study addresses this gap by conducting a global survey of surgical doctoral programs, using a structured rating scale to assess program quality. Our research questions were: (1) What is the extent of variability in PhD program requirements in Surgery internationally? (2) Can a structured scale identify high-quality programs (“excellence-PhD” or e-PhD)? (3) Are differences in program quality associated with contextual factors such as funding availability, geographic region, or university ranking? By answering these questions, we aim to provide a framework for comparing and benchmarking doctoral training, identifying best practices, and informing future efforts toward standardization and quality improvement in surgical PhD education.

## Methods

### Design

A cross-sectional survey study was designed to gather information of the content and quality of doctoral programs to obtain a PhD in surgery offered by universities around the world. The study was conducted in accordance with the Declaration of Helsinki and the reporting of this study conforms to the Checklist for Reporting Survey Studies (CROSS)^18^ (Supplemental Material) and the AAPOR reporting guidelines for survey studies.^19^ Approval of the study protocol by the Research Ethics Committee of Hospital Universitari Parc Taulí (Sabadell, Barcelona, Spain) was waived (January 16, 2024) due to the qualitative research nature of the survey study. Written informed consent was obtained from all participants.

### Participants and Setting

Participants in the study were people involved in the Surgery PhD program of all universities that belonged to the 193 countries that are member states of the United Nations.^20^ The World Directory of Medical Schools (https://search.wdoms.org/) was used to obtain the list of universities for each country. Contacts (and email addresses) were obtained through the official websites of each individual institution, which was also complemented by searching Google using the specific name of the university combined with “doctorate programs” and/or “general surgery PhD”. In some cases, names of participants were verified through personal knowledge or email with the institution. Participants were required to have an academic position or be directly involved as staff in the PhD programs in surgery at their corresponding universities and gave consent to take part in the survey. Exclusion criteria other than these specifications were not established.

### Study Procedures and Data Collection

The survey was conducted between September and October 2022 using the Google form application, in which consent to participate was a required “YES” answer. In cases in which no response was obtained, a reminder was sent after one month. The surveys were anonymous and only one response option was allowed per question. The survey invitation asked the recipient contact to complete the survey or to forward it to another faculty member qualified to respond for the doctoral program in surgery. The invitation specified that only 1 survey be completed by medical school.

On the basis of a literature search on investigating the landscape of doctoral programs in surgery, we developed an ad hoc questionnaire that included 11 items related to the doctoral program and the thesis requirements. A first box was also included where the respondent had to indicate their academic position. This questionnaire has been used in a previous study by our group.^2^ Briefly, information recorded included the duration of the program and the duration for thesis defense, financial support from the university, process of annual approval of the program progress, options for the modality of thesis dissertation (original research project or compendium of publications), type of study for an original research project, number and type of articles for the compendium of publications, journal quartile ranking, and the doctoral candidate's position in the list of authors. The journal quartile rankings were those defined in the 2022 edition of the Journal Citation Reports (JCR^®^) of the Web of Science.

In addition, responses to 7 items of the questionnaire were rated from “not relevant” to “very relevant” using an arbitrary scoring system from 0 to 5 depending on the number of possible responses to each question. The final score ranged from to 0 to 25. A score equal to or higher than 15 was considered to indicate that the doctoral program had a high academic value and was defined as an “excellence-PhD” (e-PhD).

This score was decided *ad hoc* by the research team since no validated instruments for this purpose have been developed. Importantly, this scoring system was constructed exclusively from program-level variables (duration, funding opportunities, dissertation format and requirements, number and type of publications, journal quartiles, and authorship criteria). Geographic region, socioeconomic groupings (EU, G8, G20), and university position in the Shanghai ranking^21^ (categorized by rank and divided into 1-600 and >601) were not included in the score itself; they were used only as external stratifiers for post-hoc comparisons in order to explore whether contextual factors were associated with differences in program quality.

### Statistical Analysis

Categorical variables are expressed as frequencies and percentages, and continuous variables as mean and standard deviation (SD) or median and interquartile range (IQR) (difference between the 75th and 25th percentiles). Categorical variables were compared with the chi-square test or the Fisher's exact test, and quantitative variables with the Student's *t* test, one-way analysis of variance (ANOVA), the Mann-Whitney *U* test, or the Kruskal-Wallis test according to conditions of application. Statistical significance was set at *P* < .05. The SPSS software, version 27 (IBM Corp) was used for the analysis of data.

## Results

**Figure 1.**
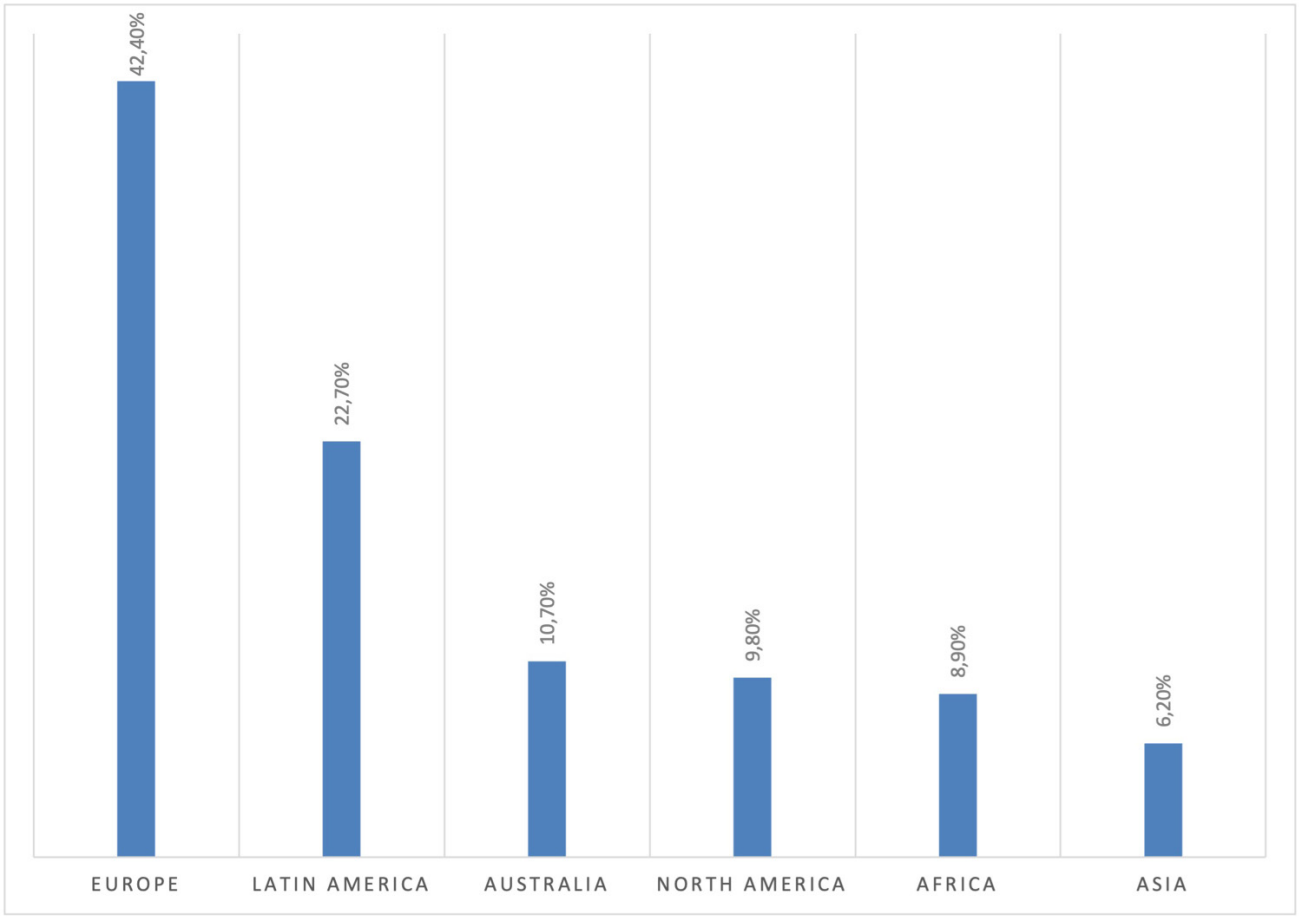
Geographical Distribution of Responses.

**Figure 2.**
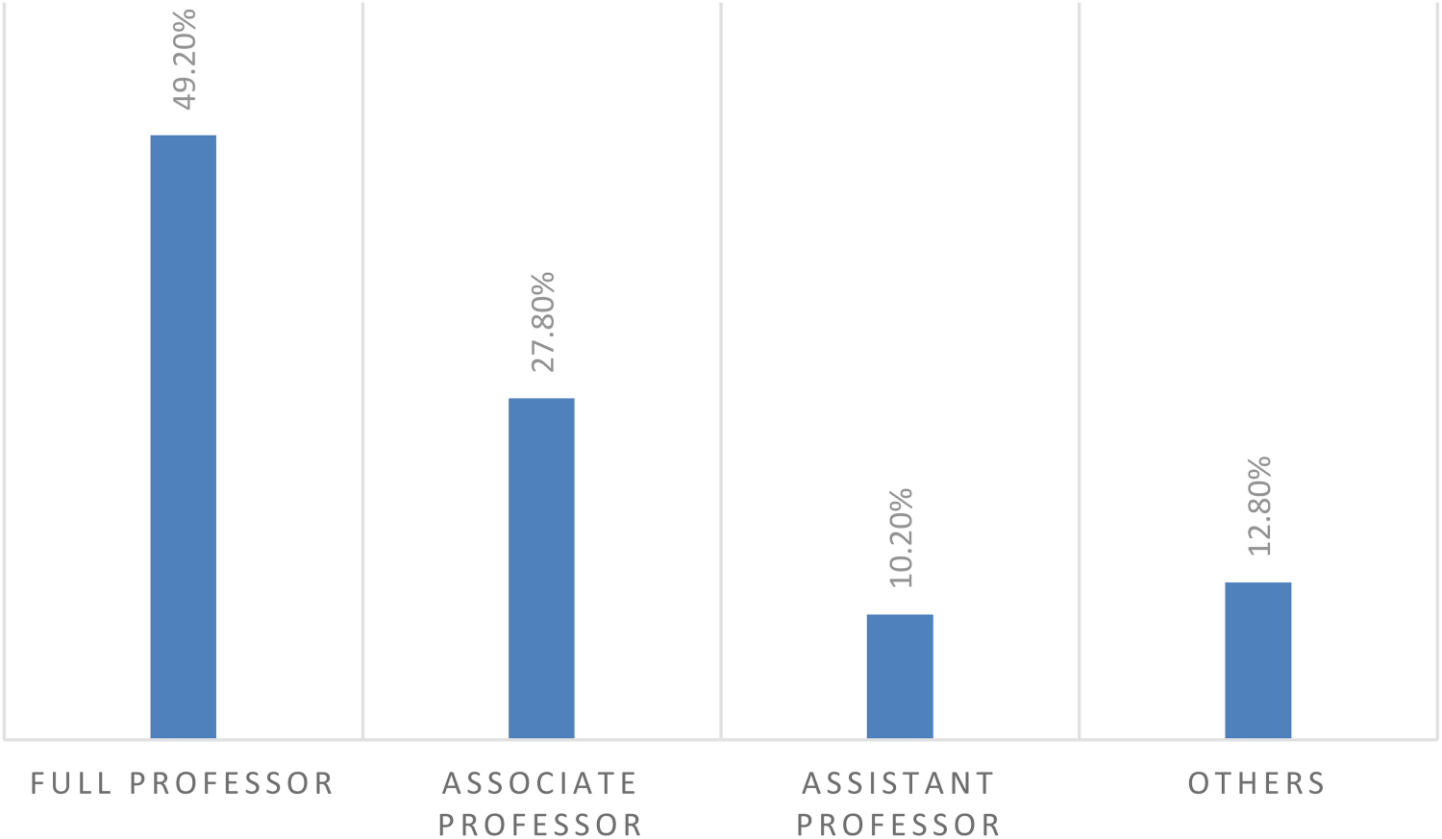
Academic Position of Respondents.

Surveys were sent to 949 surgery PhD programs and responses from 187 located in 59 countries were obtained (response rate 19.7%). The geographical distribution of responses was as follows in Figure 1 and Europe (123/290; 42.4%) provided the majority of the answers. The academic position of respondents (Figure 2) included full professor in 92 out of 187 (49.2%) cases.

### General Characteristics of the Doctoral Programs

Responses to the first 4 items of the questionnaire regarding duration of the doctoral program and funding details are shown in Table 1. The duration of full-time doctoral programs ranged between 3 and 5 years in 126 out of 187 departments (67.4%), whereas the duration of part-time programs ranged between 4 and 6 years in 53 out of 187 (28.3%). In both cases, however, a considerable number of respondents were unaware of the duration of the programs. On the other hand, there was a large variability in the time limit for presenting the dissertation and obtaining the PhD degree. In 26 out of 187 (13.9%) programs, a minimum time limit was not established or theses may be submitted at any time after matriculation in the program. In relation to funding, most universities 57 out of 187 (30.5%) did not offer any type of financial support to doctoral students.

**Table 1.** Characteristics of the Doctoral Program According to Duration and Funding.

Questions	Responses (%)^a^
Maximum duration of the full-time doctoral program, including extensions	
No limit	16 (8.6)
<2 years	8 (4.3)
3 years	47 (25.1)
4 years	45 (24.1)
5 years	34 (18.2)
≥6 years	15 (8.0)
Don’t know	22 (11.7)
Maximum duration of the part-time doctoral program, including extensions	
No limit	27 (14.4)
Part-time option is not available	32 (17.1)
<3 years	14 (7.5)
4 to 6 years	53 (28.3)
7 years	12 (6.4)
8 years	8 (4.3)
≥9 years	6 (3.2)
Don’t know	35 (18.7)
Minimum time limit for presenting the dissertation and obtaining the PhD degree	
There is no minimum limit	17 (9.1)
From matriculation at any time (first matriculation)	9 (4.8)
From the second matriculation in the program	34 (18.2)
From the third matriculation in the program	65 (34.7)
From the fourth matriculation in the program	18 (9.6)
From the fifth matriculation in the program and beyond	9 (4.8)
Don’t know	22 (11.8)
Others	13 (6.9)
Does the university offer funding to doctoral students? (allowing full-time and exclusive engagement in the research)	
Funding is not offered	57 (30.5)
Funding is offered to some doctoral students, but the aim of funding is not for the student to dedicate themselves full-time/exclusively to research	30 (16.0)
Funding is offered to all doctoral students, but the aim of funding is not for the student to dedicate themselves full-time/exclusively to research	7 (3.7)
Funding is offered to some doctoral students, with the aim of the student devoting themselves full-time/exclusively to research	40 (21.4)
Funding is offered to all doctoral students, with the aim of the student devoting themselves full-time/exclusively to research	7 (3.7)
Don’t know	15 (8.0)
Private funding	14 (7.5)
Others	17 (9.1)

^a^Total number of responses = 187.

The characteristics of the doctoral program regarding approval of the process of annual follow-up and modality of thesis dissertation are shown in Table 2. In more than half of the departments (*n* = 99), a report and approval by the tutor/director of the thesis was required for the follow-up of the research study, with an oral presentation of the project on the first year of matriculation in 41 out of 187 (22%). The doctoral dissertation was based on an original research report and/or compendium of articles on the topic of the thesis in 102 out of 187 (54.5%) departments, but a compendium of articles was accepted by only 17 (9%) and an original research report by 43 out of 187 (23%). However, the type of doctoral dissertation was not specified in 25 out of 187 (13.4%) cases.

**Table 2.** Characteristics of the Doctoral Program Regarding Approval of the Process of Annual Follow-Up and Modality of Thesis Dissertation.

Questions	Scores for Quality Assessment^a^	Responses (%)^b^
How is the annual progress of the doctoral program approved?		
There is no control, it is approved every year systematically until the presentation of the thesis / Don’t know	0	40 (21.4)
It is done administratively with the agreement of the director and/or tutor	1	35 (18.7)
A report and approval by the tutor and director is required	2	58 (31.0)
In the first year, an oral presentation and defence of the project is required. A report and approval by the tutor and director is required for the following years	3	41 (21.9)
Others	0	13 (6.9)
The PhD degree is obtained after the presentation of the doctoral dissertation before a panel, which will be based on:		
An original research project	1	43 (23.0)
An original research report and/or compendium of articles on the topic of the thesis	2	102 (54.5)
Compendium of articles on the topic of the thesis only	5	17 (9.1)
The type of research project or the compendium of articles is not specified in the doctoral program / Don’t know	0	25 (13.4)
If the modality of a compendium of articles is chosen, is a minimum number of articles that should be accepted by an academic journal specified?		
No	0	74 (39.6)
One	1	22 (11.8)
Two	2	27 (14.4)
Three	3	39 (20.9)
Four or more	4	25 (13.4)
If the modality of a compendium of articles is chosen, should these be original articles? (ie, editorials, letters to the editor, case series, research projects, book chapters, or systematic reviews are not accepted).		
No	0	77 (41.2)
At least one	1	26 (13.9)
At least two	2	25 (13.4)
All	3	59 (31.5)
If the modality of a compendium of articles is chosen, should these articles by published in journals included in the first (Q1) or second (Q2) quartile?		
They do not need to be from the first or second quartile (not specified, or there is no regulation)	0	127 (67.9)
At least one must be	1	21 (11.2)
At least two must be	2	14 (7.5)
All must be	3	25 (13.4)
If the modality of a compendium of articles is chosen, should the doctoral student be in a preferred position? (such as first author, corresponding author, or last author)		
No (not specified or there is no regulation)	0	90 (48.1)
At least in one of the articles	1	29 (15.5)
At least in two of the articles	2	32 (17.1)
In all of the articles	3	36 (19.2)

^a^Scores assigned to each variable to evaluate the quality of the doctoral program (from 0: not relevant to 3, 4, or 5: very relevant); ^b^Total number of responses = 187.

Non-randomized experimental studies and laboratory studies were required by 26 out of 187 (13.9%) of departments, but randomized experimental studies were necessary in only 4.8% (*n* = 9). In case of thesis dissertations based on a compendium of articles, at least two publications were required by 91 out of 187 (48.7%) of universities and at least three by 64 out of 187 (34.2%). All articles should be original research reports in 59 out of 187 (31.5%) departments and in 77 out of 187 (41.2%) specific conditions regarding this point were lacking. Also, in most departments 126 out of 187 (67.9%), whether articles should have been published in Q1 or Q2 journals was not specified, and in 90 out of 187 (48.1%) of cases there were no clear indications regarding the position of the doctoral student in the byline. A preferred position as first author, corresponding author, or last author was required in all publications by only 36 out of 187 (19.3%) departments.

### Quality of the Doctoral Programs

The median overall score was 8 IQR 9 (4 to 13) and a total of 24 doctoral programs out of 187 (12.8%) achieved a score ≥ 15 points) (e-PhD). However, differences in the scores of programs by continents, EU member countries, G20 countries, and university rankings of the Shanghai classifications were statistically significant (Table 3). Europe and Australia showed the highest scores as well as EU members compared to non-EU members. G8 and G20 members showed the same median score. In relation to the position of the universities in the Shanghai ranking classification, higher ranking universities showed higher scores as compared to those in lowest positions.

**Table 3.** Quality Score of the Doctoral Programs.

Variables	Number of Departments^a^	Score Median	Interquartile Range	*P* Value
Funding				
Not funded or partially funded	180	8	9 (4-13)	.96^b^
Fully funded	7	8	3 (7-10)	
Continent				
Europe	123	11	8 (7-15)	<.001^c^
North America	7	4	5 (2-7)	
Latin America	23	6	8 (2-10)	
Asia	23	5	5 (3-8)	
Africa	8	6	10 (1-11)	
Australia	3	14	3 (11-14)	
European Union (EU)				
Member country	103	11	7 (8-15)	<.001^b^
Non-member country	84	6	8 (2-10)	
G8 group				
Member country	52	6	8 (2-10)	.063^b^
Non-member country	135	9	8 (5-13)	
G20 group				
Member country	73	6	9 (1-10)	.020^b^
Non-member country	114	10.5	7 (7-14)	
Shanghai ranking universities				
1 to 200	52	11	8 (7-15)	.039^c^
201 to 400	28	9.5	8 (6-14)	
401 to 600	13	9	12 (3-15)	
601 to 800	15	10	7 (7-14)	
801 to 1000	12	9.5	10 (4-14)	
>1001	67	6	10 (1-11)	
Shanghai ranking universities				
1 to 600	93	11	9 (7-16)	.017^b^
>601	94	7	9 (4-13)	

^a^Total number = 187. ^b^The Mann-Whitney U test. ^c^The Kruskal-Wallis test.

## Discussion

The main finding of the study is the presence of a large variability at international level in the requirements of doctoral programs to obtain a PhD in surgery. Despite the existence of regulations of PhD studies at each university, the level of requirements is just defined locally rather than globally. In fact in some items of the questionnaire respondents indicated that they wither did not know the specifications or that there were no specification for that item.

The duration of doctoral programs, both part-time and full-time, is governed by the regulations in force in the particular country. As far as funding is concerned practices differ notably, ranging from programs that offer no funding at all to others that provide it systematically to all doctoral students, so that they could dedicate themselves full-time to the research project. As described in the literature, the probability of leaving the program has been related with its funding.^10^

The variability in the level of requirements of doctorate programs was also observed in the way in which the annual follow-up was evaluated. In 22% of the programs, an oral presentation of the project was necessary in the first year and subsequently an annual report approved by directors/tutors was required. In 21% of programs, however, there were no regulations in this regard.

In more than half of the departments surveyed, the thesis dissertation was based on a research project and/or a compendium of articles. The doctoral thesis obtained from a compendium of scientific articles seems to offer a guarantee of quality provided that articles had been published in prestigious peer-reviewed journals indexed the JCR^®^ database. However, we found that only 9% of doctoral programs required PhD degrees based on a compendium of published papers. In our study, 64 out of 187 (34.2%) of respondents indicated that at least three articles should be necessary for publication-based theses, but in 75 out of 187 (40%) of programs neither the minimum number of published articles nor the type of publication (such as an original article) was specified.

Moreover, other important aspects regarding authorship (preferred position of the doctoral student in the byline) and journals’ ranking in the first (Q1) or second (Q2) quartile of the JCR^®^ database appeared to be undefined in 75 out of 187 (40%) and 127 out of 187 68% of the PhD regulations, respectively. On the other hand, whether papers should be published or unpublished (eg, submitted) was not investigated. Although a collection of co-authored papers (with the doctoral student as one of the principal authors) is a common norm for doctoral dissertations in the health sciences, there is no consensus on how to partition authorship credit between PhD candidates and their coauthors.^22^ In the present questionnaire, we considered that the roles of the first author, last author, and corresponding author are relevant, but guidelines for PhD programs appear to be inconsistent as this aspect was not specified or there were no regulations in 90 out of 187 (48%) of the academic departments.

When the doctoral thesis was based on a research project, the project must be original but other characteristics are not well defined, including the type of study design according to the level of scientific evidence, which varies from descriptive observational studies to prospective randomized clinical trials.

The marked variability in the requirements of doctoral programs in surgery found in the present survey may support the assignment of different levels of demands of PhD degrees obtained at different universities. A scoring system designed to identify highly demanding programs, which may be inferred of high academic quality was used.

The highest quality scores included the modality of publication-based dissertations with the requirement of at least four articles as full research reports, published in high-impact Q1 and Q2 journals, and in which the name doctoral student has a preferred position among the authors. Although, in our opinion, the criteria of publication-based dissertations with articles published in high-impact Q1 and Q2 journals, and a preferred position of the doctoral student among the authors seems a reasonable proposal, the lack of use of these criteria does not invalidate other quality aspects of PhD programs.

If the university stipulates a research report, we think that the design should be a randomized experimental study. The higher median quality scores corresponded to doctoral program from Europe, country members of the EU, and universities on the list of the global top 200 in the 2022 Shanghai Ranking. In general, universities with higher Shanghai rankings obtained higher scores on the PhD rating scale.

The present study provides a view of the characteristics, requirements, and quality of PhD programs in surgery currently available in 187 academic departments at international level. Of note, the present results should be interpreted considering a low response rate of 20% and the large representativeness of European institutions, a limitation that may be addressed in future studies. To our knowledge, however, assessment of the variability of doctoral programs necessary to obtain a PhD in surgery on a worldwide basis has not been previously reported.

Given the findings described, standardization of the criteria for obtaining a doctorate in surgery would be useful to equal the work and ensure minimum standards of quality and excellence. Funding, research requirements, and follow-up intervals, among other factors, should be stipulated and agreed upon. In particular, as most of the responses were obtained from European universities, these results highlight the need for common policy guidelines within the European Higher Education Area (EHEA), aligned with the Bologna Process, to improve comparability and mobility of PhD-trained surgeons. Such frameworks may also inspire global initiatives to promote best practices and reduce heterogeneity in doctoral education. In relation to the variability observed, we suggest that international efforts should focus on establishing minimum standards for PhD programs in surgery. These could include: (1) research requirements, such as a minimum number of original publications in peer-reviewed Q1–Q2 journals with the candidate holding a main authorship role; (2) the provision of structured funding opportunities to allow full-time research dedication; and (3) mandatory evaluation at regular intervals (eg, annual reports and oral defenses) to monitor and ensure candidate progress. Such measures would help standardize doctoral training and guarantee minimum levels of quality and excellence worldwide.

It should be noted that the questionnaire and scoring system used in the study have not been validated and, although the internal validity of the study is not compromised by this fact, it should be mention as a limitation of the study. Moreover, a sample size calculation was not performed and the response rate depended on those participants who voluntarily and anonymously agreed to complete the survey. The lack of previous calculation of the sample size is a further limitation of the study.

## Conclusions

This survey study shows a high variability in the characteristics and type of requirements in surgery doctoral programs worldwide. Further regulations of publication-based thesis dissertations, implementation of fully funded PhD programs, and strict follow-up of the progress of research projects will probably have a notable impact on the level of quality of doctoral programs and subsequent standards in medical education.

## Supplemental Material

sj-docx-1-mde-10.1177_23821205251389679 - Supplemental material for The Absence of International Standardized Quality Criteria in Doctorate Programs in Surgery: A Survey Study Supplemental material, sj-docx-1-mde-10.1177_23821205251389679 for The Absence of International Standardized Quality Criteria in Doctorate Programs in Surgery: A Survey Study by Núria Llorach-Perucho, Manuel Pera, Eloy Espín-Bassany, Joan-Francesc Julián-Ibáñez, Juan Morote-Robles, Natalia Amat-Lefort, Álvaro Serra-Gómez, Luis Grande, Salvador Navarro-Soto and Xavier Serra-Aracil in Journal of Medical Education and Curricular Development

## References

[1] Robertson H . 8 big differences between the US and UK PhD experience. News 11 March 2020. Nature Index. Accessed September 26, 2024. https://www.nature.com/nature-index/news/eight-big-differences-between-united-states-united-kingdom-phd-experience-researcher-grad-school

[2] Serra-Aracil X Armengol Carrasco M Morote Robles J , et al. Is there the same requirement to obtain the PhD degree in all the departments of surgery of the Spanish universities? Cir Esp (Engl Ed). 2022;100(8):496-503. doi:10.1016/j.cireng.2022.05.018 35597418

[3] Real Decreto 99/2011, de 29 de enero, por el que se regulan las enseñanzas oficiales de doctorado. BOE núm 35, de 10/02/2011. Accessed September 26, 2024. https://www.boe.es/eli/es/rd/2011/01/28/99/con

[4] Belavy DL Owen PJ Livingston PM . Do successful PhD outcomes reflect the research environment rather than academic ability? PLoS One. 2020;15(8):e0236327. doi:10.1371/journal.pone.0236327 PMC7406039 32756557

[5] Corsini A Zaider I Visentin F . What makes a productive Ph.D. student? Res Policy. 2022;51(10):104561. doi:10.1016/j.respol.2022.104561

[6] Ajjawi R Crampton PES Rees CE . What really matters for successful research environments? A realist synthesis. Med Educ. 2018;52(9):936-950. doi:10.1111/medu.13643 30043516 PMC6120529

[7] Seckin OC Varol O . Academic support network reflects doctoral experience and productivity. EPJ Data Sci. 2022;11(1):20. doi:10.1140/epjds/s13688-022-00369-z35371907

[8] Singh P Aggarwal R Darzi A . Review of selected national surgical curricula: quantity is not the sole marker of quality. J Surg Educ. 2014;71(2):229-240. doi:10.1016/j.jsurg.2013.07.015 24602715

[9] Tahir M Rahman U Gulati A . An international comparison of competency-based orthopaedic curricula and minimum operative experience: review article. Int J Surg. 2021;94:106125. doi:10.1016/j.ijsu.2021.106125 34592430

[10] Joshi A Borraez-Segura B Anwer M , et al. An international collaborative study on surgical education for quality improvement (ASSURED): a project by the 2017 international society of surgery (ISS/SIC) travel scholars international working group. World J Surg. 2020;44(5):1400-1411. doi:10.1007/s00268-019-05342-y 31907571

[11] Rickard J . Systematic review of postgraduate surgical education in low- and middle-income countries. World J Surg. 2016;40(6):1324-1335. doi:10.1007/s00268-016-3445-x 26902628

[12] Elmaraghi S Park KM Rashidian N , et al. Postgraduate surgical education in east, central, and Southern Africa: a needs assessment survey. J Am Coll Surg. 2023;236(2):429-435. doi:10.1097/XCS.0000000000000457 36218266

[13] Wurdeman T Menon G Meara JG Alkire BC . A country-level comparison of access to quality surgical and non-surgical healthcare from 1990–2016. PLoS One. 2020;15(11):e0241669. doi:10.1371/journal.pone.0241669 PMC7608906 33141856

[14] Ludwig J Jakobsen RB Charles YP , et al. What it takes to become an orthopaedic surgeon: a comparison of orthopaedic surgical training programmes in 10 countries focusing on structure and fellowship requirements. Int J Surg. 2021;95:106150. doi:10.1016/j.ijsu.2021.106150 34715383

[15] Ziemann E Oestmann JW . Publications by doctoral candidates at Charité University Hospital, Berlin, from 1998-2008. Dtsch Arztebl Int. 2012;109:333-337. doi:10.3238/arztebl.2012.0333 22679453 PMC3369306

[16] Rømer T Hansen MT Helge JW . An analysis of the productivity and impact of clinical PhD theses from the University of Copenhagen. Dan Med J. 2020;67(5):A12190731. 32351199

[17] Ostriker JP Holland PW Kuh CV Voytuk JA . Committee to assess research-doctorate programs; National Research Council. Accessed September 26, 2024. https://www.nap.edu/catalog/12850.html

[18] Sharma A Minh Duc NT Luu Lam Thang T , et al. Consensus-based checklist for reporting of survey studies (CROSS). J Gen Intern Med. 2021;36(10):3179-3187. doi:10.1007/s11606-021-06737-1 33886027 PMC8481359

[19] Pitt SC Schwartz TA Chu D . AAPOR Reporting guidelines for survey studies. JAMA Surg. 2021;156(8):785-786. doi:10.1001/jamasurg.2021.0543 33825811

[20] United Nations . Member states. Accessed July 8, 2021. https://www.un.org/en/about-us/member-states

[21] Shanghai Ranking . 2022 academic ranking of world universities. Accessed June 13, 2023. https://www.shanghairanking.com/rankings/arwu/2022

[22] Hagen NT . Deconstructing doctoral dissertations: how many papers does it take to make a PhD? Scientometrics. 2010;85(2):567-579. doi:10.1007/s11192-010-0214-8 20949112 PMC2943069

